# Distribution and breeding sites of *Aedes aegypti* and *Aedes albopictus* in 32 urban/peri-urban districts of Mozambique: implication for assessing the risk of arbovirus outbreaks

**DOI:** 10.1371/journal.pntd.0006692

**Published:** 2018-09-12

**Authors:** Ana Paula Abílio, Gastão Abudasse, Ayubo Kampango, Baltazar Candrinho, Salomão Sitoi, Jacinta Luciano, Dário Tembisse, Samira Sibindy, António Paulo Gouveia de Almeida, Gabriela Azambuja Garcia, Mariana Rocha David, Rafael Maciel-de-Freitas, Eduardo Samo Gudo

**Affiliations:** 1 National Institute of Health, Ministry of Health, Maputo, Mozambique; 2 National Malaria Control Program, National Directorate of Public Health, Ministry of Health, Maputo, Mozambique; 3 GHTM, Institute of Hygiene and Tropical Medicine, Universidade Nova de Lisboa, Portugal; 4 Laboratório de Transmissores de Hematozoários, Oswaldo Cruz Institute, Fiocruz, Rio de Janeiro, Brazil; Centers for Disease Control and Prevention, UNITED STATES

## Abstract

**Background:**

*Aedes*-borne arboviruses have emerged as an important public health problem worldwide and, in Mozambique, the number of cases and its geographical spread have been growing. However, information on the occurrence, distribution and ecology of *Aedes aegypti* and *Ae*. *albopictus* mosquitoes remain poorly known in the country.

**Methods:**

Between March and April 2016, a cross-sectional study was conducted in 32 districts in Mozambique to determine the distribution and breeding sites of *Ae*. *aegypti* and *Ae*. *albopictus*. Larvae and pupae were collected from a total of 2,807 water-holding containers using pipette, dipper, funnel and sweeping procedures, depending on the container type and location. Both outdoor and indoor water-holding containers were inspected. The immature forms were reared to adults and the identifications of the mosquito species was carried out with a stereomicroscope using a taxonomic key.

**Results:**

*Aedes aegypti* was found in every district sampled, while *Ae*. *albopictus* was only found in Moatize district, situated in Tete Province in the central part of the country. Six hundred and twenty-eight of 2,807 (22.4%) containers were positive for *Ae*. *aegypti* but only one (0.03%) was positive for *Ae*. *albopictus*. The Container Index (CI) of *Aedes* was highest in densely populated suburban areas of the central region (260/604; 43.0%), followed by suburban areas in northern areas (228/617; 36.9%) whilst the lowest proportion was found in urbanized southern areas (140/1586; 8.8%). The highest CI of *Aedes* was found in used tires (448/1268; 35.3%), cement tanks (20/62; 32.3%) and drums (21/95; 22.1%).

**Conclusion:**

Data from our study showed that *Ae*. *aegypti* is present nation-wide, since it occurred in every sampled district, whilst *Ae*. *albopictus* had a limited distribution. Therefore, the risk of transmission of dengue and chikungunya is likely to have been underestimated in Mozambique. This study highlights the need for the establishment of a national entomological surveillance program for *Aedes spp*. in Mozambique in order to gain a better understanding about vector bionomics and to support the development of informed effective vector control strategies.

## Author summary

Dengue, chikungunya and Zika are a group of rapidly spreading mosquito-borne diseases worldwide. These arboviral diseases have received increasing attention in Mozambique as a consequence of recent dengue outbreaks, which occurred in the northern region. There has also been an increase in the number of cases of chikungunya reported in the country. Additionally, earlier evidence obtained from neutralizing antibodies against Zika revealed an overall prevalence of 4% in 249 individuals (142 adults and 107 children) sampled from 22 localities across Mozambique in the 1950’s. These arboviruses are primarily transmitted by the bites of infected *Aedes* (*Stegomyia*) females, especially *Ae*. *aegypti* and *Ae*. *albopictus*. However, data on the distribution and the bio-ecology of both *Aedes* species are scarce. This lack of information is a major barrier for the implementation of public health interventions to prevent *Aedes*-borne arbovirus infections. In this study, we investigated the distribution and abundance of *Ae*. *aegypti* and *Ae*. *albopictus* in 32 districts of Mozambique. We found *Ae*. *aegypti* in every district sampled, although with heterogeneous abundance, while *Ae*. *albopictus* had limited occurrence. *Aedes aegypti* breeding sites varied among districts. The predominant containers were used tires, cement tank and drums, all present at high densities in central and northern Mozambique. This is the first study that investigates the distribution of breeding sites and abundance of *Aedes spp*. in a large number of districts in Mozambique and provides relevant baseline data for the establishment of a vector surveillance and control interventions for arboviruses in the country.

## Introduction

Dengue, chikungunya and Zika are among the most important mosquito-transmitted viruses worldwide. Their global burden of these diseases has increased rapidly in the last decades [1, 2]. An estimated 390–500 million cases of dengue occur every year [1, 3]. Zika was declared a public health emergency of international concern in February 2016 [4], whilst Chikungunya virus has caused massive and severe outbreaks worldwide over the last decade [5–7]. The spread of these viruses follows the distribution of the primary vector, *Aedes aegypti* [8]. *Ae*. *aegypti* originated in Africa, but is now found in more than 120 countries worldwide [8–10], including countries situated in temperate regions [11–13]. Additionally, *Ae*. *albopictus* which is considered to be a potential vector of several arboviruses, has also expanded its geographical distribution [14, 15]. In 2015 its presence was confirmed in Maputo, Mozambique’s capital [14, 15].

Sub-Saharan Africa is at particularly high risk of occurrence and spread of *Aedes* transmitted pathogens due to its climate and environmental conditions. Recent studies presented evidence of arboviruses in Mozambique, such as the recent confirmation of a DENV-2 outbreak in 2014 during which a total of 100 confirmed/probable cases were reported [16]. Subsequently, the endemic circulation of DENV-2 was demonstrated in 2015–2016, from a total of 21 PCR-positive samples detected in northern Mozambique [17]. Anti-CHIKV IgG antibodies were found in 26.4% of the samples from a cohort of convalescent patients with acute febrile symptoms in Maputo city in 2013 and a case of severe chikungunya infection was reported in the Northern region of the country in 2014 [18]. These findings of arbovirus circulation in the country provide convincing evidence that transmission risk might be higher than expected. Several biotic and abiotic factors might also enhance the transmission risk of *Aedes*-borne arboviral diseases in Mozambique. The country is the third most vulnerable to extreme climate events, such as floods and droughts in Sub-Saharan Africa [19]. The frequency of unusually long periods of droughts have increased in the last decades leading people to opt for water storage practices which might increase the number of sites suitable for *Aedes spp* larvae [20]. In addition the rate of unplanned urbanization in Mozambique is high, favoring the presence of high population densities with associated artificial breeding sites for the mosquitoes [21]. Field studies of *Aedes* populations of sub-Saharan Africa are mostly from East, Central or West Africa [22–32] and little data is available for the Southern region of Africa. In particular in Mozambique [33], with the exception of an exploratory study conducted in four districts during a dengue outbreak in 2014 [34], there has been no systematic study concerning the distribution of *Aedes spp* populations. This is a barrier for the implementation of preventive and control interventions. This report, therefore, describes the results of the first country-wide survey of the density, distribution and breeding sites of *Aedes spp* in Mozambique.

## Methods

### Study area

Mozambique is situated in southeast coast of Africa with 2,515 km of coastline, and an estimated population of 27 million inhabitants [35]. The climate is tropical with two distinct seasons, namely; the rainy season from November-April and dry season from May-October. The average humidity ranges between 70–80%, with highest values being reported in Central and North regions. The average annual air temperature varies between 20°C in the South to 26°C in Northern regions.

### Ethics statement

The study was approved by the Mozambican National Bioethics Committee (Ref #: 05/CNBS/2016). Oral consent to examine potential breeding habitats was obtained from the head of the household.

### Sampling design and households selection

A cross-sectional study was conducted between March 19 and April 30, 2016, during the rainy season, in a total of 32 districts. Households were selected using a sampling approach stratified into three stages. The first stage involved the selection of all the eleven provinces of Mozambique to ensure that every province is represented in this survey. In each province, three districts and in each district, one village or neighbourhood were selected as a second stage, on the basis of the following criteria: i) occurrence of confirmed dengue cases in the preceding months or years, and ii) climatic and socio-demographical factors (human population density and degree of urbanization) considered suitable for the occurrence and establishment of dengue vectors. The most populated and urbanized village or neighbourhood was preferentially chosen.

A spatial sampling procedure oriented to clusters of households was adopted to select households. A cluster was considered as a geographical area comprising between 10–20 households located within a radius of 50–100 metres. The selection of a household cluster was carried out following the procedure described by Troyo *et al*. [36]. According to this procedure, an administrative map of each village/neighbourhood was obtained using Google Earth Pro v. 7.3.0 (Google Inc., USA). Then, grid cells of 10km^2^ of the area were drawn on the map. The number of grid cells varied according to the size of the region. Grids were numbered starting from the cell on the upper left corner of the map. Then, a random sample of three 10km^2^ area grids was selected for the household cluster survey. In each of these grids, three clusters comprising 10–20 households were selected, based on the accessibility of the location. The clusters were at least 400 metres apart, considered to be the maximum distance of *Ae*. *aegypti* flight [37], to reduce the likelihood of pseudoreplication. A household was defined as a single unit of accommodation (individual household or an apartment) including the surrounding enclosure/compounds.

### Entomological survey

In every household, intra and peridomestic breeding sites were inspected for the presence of immature stage (larva and pupa) of *Ae*. *Aegypti* and *Ae*. *albopictus*. All selected households were assessed indoors and outdoors. We considered as outdoors any place outside the rooms, but inside the enclosure/compound, including the rooftop, while any place inside the household was classified as indoors. The immature stages were sampled in all water holding containers following standard operating procedures for *Ae*. *aegypti* [38]. Containers were classified according to the presence of larvae (positive/negative). For small containers, the total number of larvae and pupae (as well as pupa carcasses) were collected using pipettes, whereas for containers ≥ 25 litres in volume or wells, the funnel and sweeping-net technique and dipper (500 μm of mesh diameter) were used [38, 39] and ten dips and sweeps were performed per container. Larvae were transported to the insectary and reared to adults under controlled environmental conditions of temperature (27°C ± 2°C). Adults were morphologically identified using the taxonomical key of Huang [40]. The identification of specimens was double checked by two-experienced entomologists. The field team at each province comprised four entomologists, two from the central level and two from the provincial level.

### Mosquitoes collection, transportation, preservation and morphological identification

Water holding containers were categorized according to the type of container. All information related to each container including the presence of *Aedes spp*., and whether immature stages were sampled as larvae or pupae, was recorded in a field form. Immature forms were collected using pipette or dipper net (5 x 7 cm, 500 μm mesh) depending on container type and its location in the household [35]. All larvae and pupae were stored in a labeled specimen bottle and transported to local insectaries for growth until adult stage according to the standard procedures for rearing mosquitoes [51]. Upon adult emergence, mosquitoes were sacrificed and preserved on a 1.5 ml tube containing silica gel. All preserved samples were transported to the Medical Entomology Laboratory (ENTMED) at National Institute of Health (INS) in Maputo for morphological identification of the *Aedes* species under a stereomicroscope using a taxonomic key [41].

### Data analysis

Data were entered into a database developed using Microsoft Excel 2013 imported into Stata 13 for descriptive data analysis to determine the frequencies and distribution of *Ae*. *aegypti* and *Ae*. *albopictus*. The container index (CI) was determined using the following formula: CI = Total n° of positive container / Total n° of water−holding containers ×100% [42]. The spatial variation of CI estimates for each region was visualized in maps using ArcGIS 10.2 Software (ESRI Inc, Redlands, CA), were used to produce maps of occurrence.

## Results

### Geographical distribution of *Aedes spp*.

**Fig 1:**
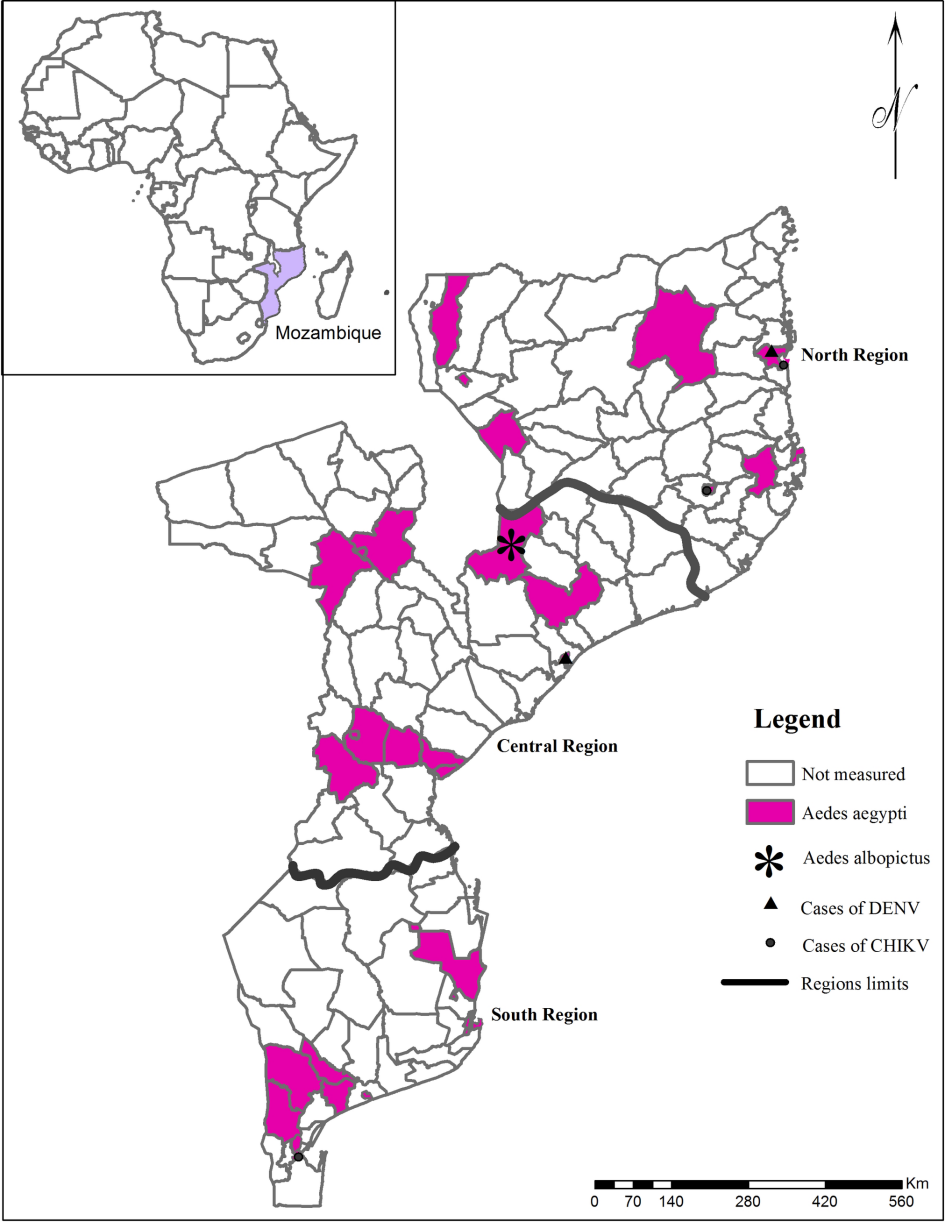
Map of Mozambique highlighting the three main regions of the country, and the geographical locations of the 32 districts studied.

A total of 2,807 water-holding containers were inspected of which 628 (22.4%) were positive for *Ae*. *aegypti*. *Aedes albopictus* was only found in a single breeding site located at Moatize district (Central region), which was also positive for *Ae*. *aegypti* (Fig 1).

Pink coloured areas depict those districts where *Ae*. *aegypti* breeding sites were found. *Aedes aegypti* was found in all sampled districts. The legend key (*) indicates the only district where *Ae*. *albopictus* was found in this survey.

**Fig 2:**
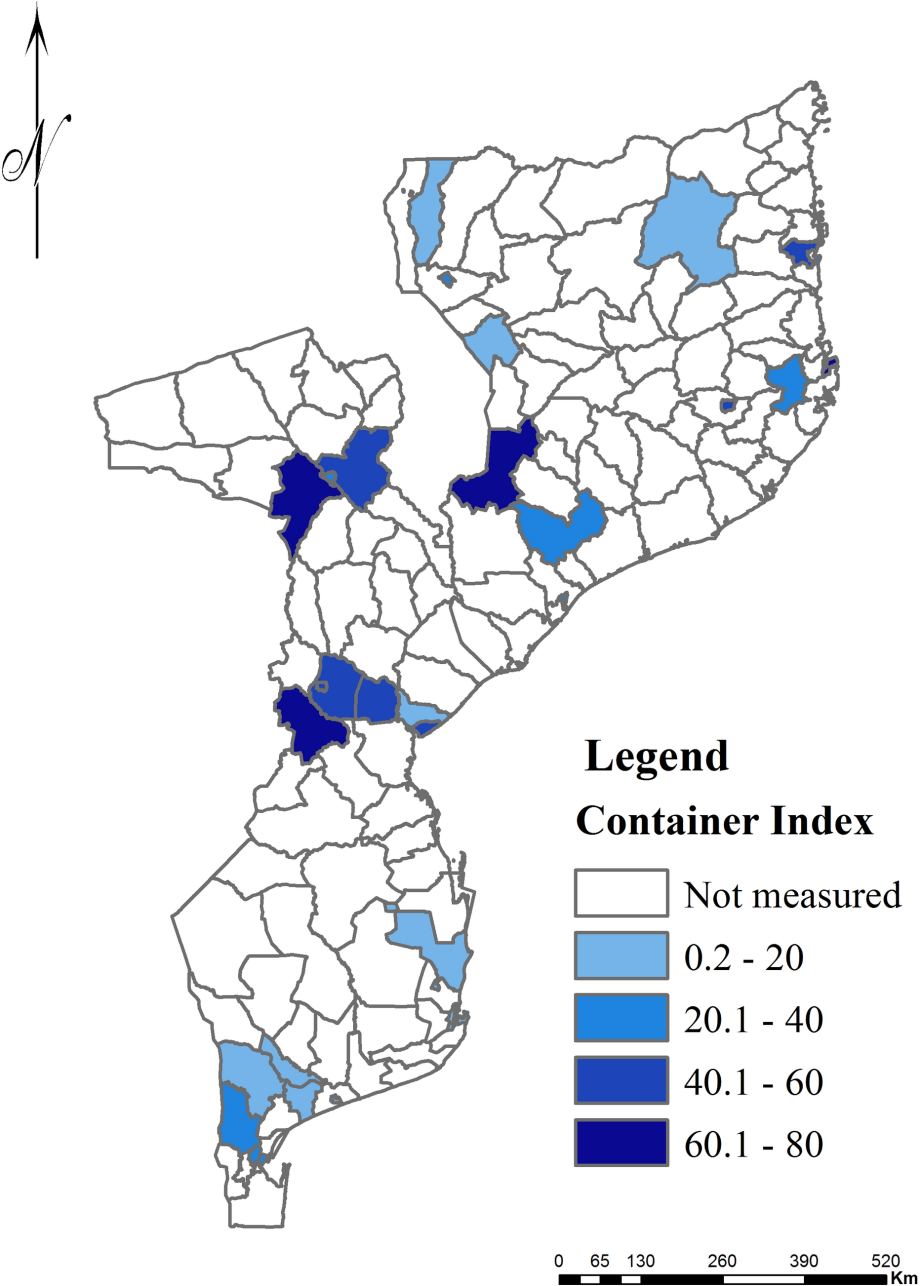
Infestation of *Aedes aegypti*, expressed as container index (CI), in 32 districts surveyed between March and April 2016.

The Container index (CI) of *Aedes spp*. was higher in the Central region (43.6%; 260/596), followed by the North (36.9%; 228/617), whilst the lowest CI was found in the South region (8.7%; 140/1594) (Fig 2).

In the Northern region, the highest *Ae*. *aegypti* CI at the Province level was reported in Nampula (49.4%; 158/320), followed by Cabo Delgado (24.3%; 28/115) and Niassa (23.1%; 42/182) (Table 1). The districts of Nacala Porto (CI = 68.1%; 47/69) and Nampula city (CI = 46.7%; 78/167) in Nampula Province, and Pemba Metuge (CI = 42.8%; 9/21), in Cabo Delgado Province exhibited the highest infestation levels of *Ae*. *aegypti* (Table 1).

**Table 1:** Presence of larvae/pupae of *Aedes* (*Stegomyia*) *spp*. per container inspected stratified by region, province, district and neighborhood, March-April 2016.

Region and Province		District	Neighborhood	*# Inspected* *n*	*# Positive*, *n (%)*	
					*Ae*. *aegypti*	*Ae*. *albopictus*
Total				2807	628 (22.4)	1 (0.03)
**Northern region**				**617**	**228 (36.9)**	
	***Cabo Delgado***			***115***	***28 (24*.*3)***	
		Pemba Metuge	3 de Fevereiro	21	9 (42.8)	
		Motepuez	Miringe	59	7 (11.8)	
		C. Pemba	Natiti	35	12 (34.2)	
	***Nampula***			***320***	***158(49*.*4)***	
		Nacala porto	Triangulo	69	47 (68.1)	
		Monapo	Topelane	84	33 (39.3)	
		C. Nampula	Muahivire	167	78 (46.7)	
	***Niassa***			***182***	***42 (23*.*1)***	
		C. Lichinga	Nzinje	112	31 (27.7)	
		Lago	Sanjala	36	6 (16.7)	
		Mandimba	Bairro Central	34	5 (14.7)	
**Central region**				**604**	**260 (43.0)**	**1 (0.2)**
	***Manica***			***200***	***107 (53*.*5)***	
		C. Chimoio	7 de Abril	84	47 (55.9)	
		Gondola	Josina Machel	58	25 (43.1)	
		Sussundenga	Nhamizara	58	35 (60.3)	
	***Sofala***			***138***	***53(38*.*4)***	
		C. Beira	Munhava	75	33 (44.0)	
		Dondo	Nhamayabue	23	3 (13.0)	
		Nhamatanda	3° Bairro	40	17 (42.5)	
	***Tete***			***52***	***24 (46*.*2)***	***1 (1*.*9)***
		C. Tete	Filipe S. Magaia	22	7 (31.8)	
		Moatize	25 de Setembro	12	6 (50.0)	1 (8.3)
		Changara	Nhalicune	18	11 (61.1)	
	***Zambézia***			***214***	***75 (35*.*0)***	
		Mocuba	Marananeulo	63	20 (31.7)	
		Milange	25 de Junho	53	33 (62.3)	
		C. Quelimane	Floresta	98	22 (22.4)	
**Southern region**				**1586**	**140 (8.8)**	
	***Gaza***			***396***	***52 (13*.*1)***	
		Xai-Xai	P. Lumumba	255	40 (15.7)	
		Chokwe	1° Bairro	73	7 (9.6)	
		Bilene	6° Bairro	68	5 (7.4)	
	***Inhambane***			***865***	***25 (2*.*9)***	
		Massinga	7 de Setembro	233	7 (3.0)	
		C. Inhambane	Chalambe 2	487	1(0.2)	
		Maxixi	Chalambe 1	145	17 (11.7)	
	***Maputo Cidade***			***40***	***15 (37*.*5)***	
		Kanfumo	Malhangalene B	36	13 (36.1)	
		Kamachaquene	Polana Caniço	4	2 (50.0)	
	***Maputo Província***			***285***	***48 (16*.*8)***	
		Magude	Ricatlana	171	10 (5.8)	
		Matola	Infulene	96	29 (30.2)	
		Muamba	Cimento	18	9 (50.0)	

Regarding the Central region, the highest *Ae*. *aegypti* CI was registered in Manica (53.5%; 107/200), followed by Tete (46.2%; 24/52) and Sofala (38.4%; 53/138) Provinces. The lowest CI was found in Zambézia Province (35.0%; 75/214). The highest *Ae*. *aegypti* infestation levels were found in Milange district (CI = 62.3%; 33/53) in Zambézia Province, Changara district (CI = 61.1%; 11/18) in Tete Province and Sussundenga district (CI = 60.3%; 35/58) in Manica Province.

In South Mozambique, the highest CI was reported in Maputo city (37.5%; 15/40), followed by Maputo (16.8%; 48/285) and Gaza (13.1%; 52/396) Provinces. The lowest CI was reported in Inhambane Province (2.9%; 25/863). The districts with highest *Ae*. *aegypti* CI in the South were Kamachaquene (50.0%; 2/4) and Kanfumo (36.1%; 13/36) in Maputo city and Matola district (30.2%; 29/96) in Maputo Province (Table 1).

### Breeding sites of *Ae*. *aegypti* and *Ae*. *albopictus*

The types of container in which larvae of *Ae*. *aegypti* were found is shown in Table 2. Used tires were the most frequent type of containers, followed by flower pots, jar/pots, cement tanks, buckets, disposed cans and bottles. A total of 2,807 potential breeding containers sub-divided into 9 different groups were sampled. The highest *Ae*. *aegypti* immature stages positivity rates were found in used tires (35.3%; 448/1268), cement tanks (32.3%; 20/62) and drums (22.1%; 21/95). On the other hand, cans (9.5%; 14/146), bottles (9.4%; 7/74) and flower pots (6.3%; 36/576) had a lower infestation (Table 2). The *Ae*. *albopictus* larvae found Moatize district, Tete Province came from a used tire.

**Table 2:** Presence of larvae/pupae of *Aedes* (*Stegomyia*) *spp*. in different breeding sites stratified by region and province, March-April 2016.

Region/Province		Total inspected (n)	Total positive, n (%)	Number of positive breeding sites/Number of total breeding sites inspected (%)								
				Used tires	Pots	Drums	Cement tanks	Buckets	Cans	Bottles	Flower pots	Plastic containers
**TOTAL**		**2807**	**628 (22.4)**	**448/1268 (35.3)**	**21/122 (17.2)**	**21 /95 (22.1)**	**20/62 (32.3)**	**17/156 (10.9)**	**14/146 (9.5)**	**7/74 (9.4)**	**34/576 (5.9)**	**46/308 (14.9)**
**Northern**	**Total**	**617**	**228 (36.9)**	**147/ 290 (50.7)**	**14/38 (36.8)**	**6/17 (35.3)**	**10/20 (50.0)**	**13/52 (25.0)**	**9/81 (11.1)**	**6/52 (11.5)**	**8/23 (34.8)**	**15/44 (34.1)**
	Cabo Delgado	115	28 (24.3)	9/18 (50.0)	1/1 (100.0)	1/1 (100.0)	7/10 (70.0)	4/18 (22.2)	1/21 (4.8)	1/29 (3.4)	2/8 (25.0)	2/9 (22.2)
	Nampula	320	158 (49.4)	110/164 (67.1)	12/ 30 (40.0)	5/12 (41.7)	0/1 (0.0)	9/28 (32.1)	5/42 (11.9)	5/23 (21.7)	-	12/20 (60.0)
	Niassa	182	42 (23.1)	28 /108 (25.9)	1/7 (14.3)	0/4 (0.0)	3/9 (33.3)	0/6 (0.0)	3/18 (16.7)	-	6/15 (40.0)	1/15 (6.7)
**Central**	**Total**	**604**	**260 (43.0)**	**203/439 (46.2)**	**1/22 (4.5)**	**10/14 (71.4)**	**1/3 (33.3)**	**0/1 (0.0)**	**4/11 (36.4)**	**1/9 (11.0)**	**26/81 (32.0)**	**14/24 (58.3)**
	Manica	200	107 (53.5)	81/154 (52.6)	1/1 (100.0)	9/10 (90.0)	1/2 (50.0)	-	-	1/9 (11.0)	-	14/24 (58.3)
	Sofala	138	53 (38.4)	53/138 (38.4)		-	-	-	-	-	-	-
	Tete	52	25 (48.1)	21/43 (48.8)	-	1/4 (25.0)	0/1 (0.0)	0/1 (0.0)	1/1 (100.0)	-	2/2 (100.0)	-
	Zambézia	214	75 (35.0)	48/104 (46.2)	0/21 (0.0)	-	-	-	3/10 (30.0)	-	24/79 (30.4)	-
**Southern**	**Total**	**1586**	**140 (8.8)**	**98/539 (18.2)**	**6/62 (9.7)**	**5/64 (7.8)**	**9/39 (23.0)**	**4 /103 (3.9)**	**1/54 (1.9)**	**0/13 (0.0)**	**0/472 (0.0)**	**17/240 (7.1)**
	Gaza	396	52 (13.1)	38/145 (26.2)	1/13 (7.7)	1/23 (4.3)	0/5 (0.0)	1/37 (2.7)	1/40 (2.5)	-	-	10/133 (7.5)
	Inhambane	865	25 (2.9)	14/193 (7.3)	3/16 (18.8)	2/24 (8.3)	2/15 (13.3)	3/44 (6.8)	0/12 (0.0)	0/13 (0.0)	0/467 (0.0)	1/81 (1.2)
	Maputo Cidade	40	15 (37.5)	8/29 (27.6)	-	2/4 (50.0)	5/7 (71.4)	-	-	-	-	-
	Maputo Província	285	48 (16.8)	38/172 (22.1)	2/33 (6.1)	0/13 (0.0)	2/12 (16.7)	0/22 (0.0)	0/2 (0.0)	-	0/5 (0.0)	6/26 (23.1)

## Discussion

Arboviruses are spreading at an alarming pace across the world and a growing fraction of them have been reported in recent years in Mozambique [8, 16, 18, 43, 44]. Data on the distribution and ecology of anthropophilic *Aedes* mosquito species in the country remains limited. Previous records from the 1960’s reported the presence of *Ae*. (*Stegomyia*) species in Northern to Southern regions, with highest densities in coastal areas [45]. However, the distribution may have changed.

*Aedes aegypti* were collected in every district sampled, which explains the transmission of DENV, CHIKV and others arbovirus in many parts of Mozambique [16–18, 43, 44, 46]. Using mathematical modeling the heterogeneity of abundance and distribution of *Ae*. *aegypti* shown in the present study has previously been suggested by Kraemer and others [8]. Similar findings were observed in Cameron [45] and in a prior study conducted in four cities of Mozambique in 2014 [34]. Thus, the risk of arbovirus transmission is also likely to be heterogeneous across the country, suggesting that vector control activities should prioritize the Central and Northern regions, the regions with higher *Ae*. *aegypti* infestation levels.

The lower abundance of *Ae*. *aegypti* in the South might be due to lower amount of rainfall [47, 48], relatively good environmental sanitation and a consistent water supply system, which reduces number of putative *Ae*. *aegypti* and *Ae*. *albopictus* breeding sites. In contrast, the high CI in Northern Mozambique may be due to the high annual precipitation [48], a poor water supply system (leading to an increase in water storage containers) and poor environmental sanitation, which increases the number of putative breeding sites such as, disposed cans and abandoned used tires.

Our results are in accordance with a preliminary investigation conducted in four districts in 2014 in Mozambique [34] and could explain why most of the arbovirus outbreaks reported so far occurred in the Northern region [16, 18, 46]. A similar pattern has been observed for malaria Southern regions having, lower prevalence rates than Central and North regions of the country [48, 49].

It is well known that unplanned urbanization represents an important driver of anthropophilic *Aedes spp*. expansion in sub-Saharan Africa [50]. Increasing urbanization is only likely to exacerbate the problem. According to the World Urbanization Prospect report, the urban population in Mozambique rose from 7.0% in 1970 to 32.8% in 2017 and it is predicted to be 50.0% by 2050 [51]. It therefore becomes increasingly important that control and monitoring starts soon.

*Aedes albopictus* was only found in Moatize district, in Tete Province, in the Central region. Our data, together with a recent report by Kampango and Abílio [15], who initially described the presence of *Ae*. *albopictus* in Mozambique in the south of the country, suggests that it may have already invaded and be successfully established in other areas of the country. The potential spread of *Ae*. *albopictus* throughout the country raises serious concerns, since it is a possible vector of at least 22 viruses affecting humans, including dengue, chikungunya, Zika, yellow fever and Japanese encephalitis virus [45, 52]. The geographical distribution of *Ae*. *albopictus* worldwide has expanded over the past three decades, with several countries reporting its presence for the first time [23–25, 53–56]. Climate change has been pointed out as a major determinant of *Ae*. *albopictus* expansion [11, 57]. Additional research is urgently needed for a better understanding of the ecological features of *Ae*. *albopictus* under local conditions.

The present survey showed that the preferred breeding site of *Ae*. *aegypti* were used tires, cement tanks and drums. This was not surprising, considering that *Ae*. *aegypti* is highly synanthropic. Old tires are commonly used in Mozambique for fencing in peri-urban and rural households, to weigh down the tin sheeting used for roofing material in some houses and to control soil erosion [34]. Furthermore, used tires are frequently sold along the main public highways, where they usually remain unattended and exposed to rainfall and sunlight for long periods. Cement tanks and drums are the most frequently found water-storage containers in communities with intermittent or deficient water supplying. Data from Cameroon, India and Vietnam [45, 58–60] also showed that water storages for domestic use in cement tanks and drums are among the most productive breeding sites of *Aedes* mosquitoes.

Thus, *Ae*. *aegypti* and *Ae*. *albopictus* control programs should concentrate their interventions on the education and engagement of residents in appropriate use and disposal of old tires and covering of water drums and tanks.

Since Mozambique has a well established sentinel surveillance system for malaria vectors, we recommend that *Aedes* surveillance be integrated into the existing surveillance system for malaria vectors that is being carried out in urban and rural areas of the country. The surveillance for *Aedes* should be enhanced to urban areas where *Ae*. (*Stegomyia*) mosquitoes are more frequent, in order to ensure its sustainability and optimize use of scarce resources.

Although we were only able to undertake samples from 32 out of the 152 districts of Mozambique ours remains the largest study conducted so far in the country. Our results indicate that *Ae*. *Aegypti* is present in all regions of the country with, therefore, a risk of dengue, Zika and chikungunya transmission in urban areas.

In conclusion, we found that *Ae*. *aegypti* has heterogeneous distribution throughout Mozambique. The mosquito is likely to be present throughout the country, enhancing the risk of dengue, chikungunya and Zika transmission. *Aedes albopictus*, another potential vector of these arboviruses, may have a more limited distribution. Further systematic studies are required to determine the degree of ecological association between these two vectors, as well as their contribution in the arboviruses transmission in the country. A national surveillance system for *Aedes spp*. in Mozambique is required.

## Supporting information

S1 File (XLSX)
